# The role of spatial and temporal information in biological motion perception

**DOI:** 10.2478/v10053-008-0006-3

**Published:** 2008-07-15

**Authors:** Joachim Lange, Markus Lappe

**Affiliations:** 1 Department of Psychology II, University of Muenster, Germany; 2 F.C. Donders Centre for Cognitive Neuroimaging, Radboud University, Nijmegen, The Netherlands

**Keywords:** biological motion, model, task dependency, event-related potentials

## Abstract

Point-light biological motion stimuli provide spatio-temporal information about the structure of the human body in motion. Manipulation of the spatial structure of point-light stimuli reduces the ability of human observers to perceive biological motion. A recent study has reported that interference with the spatial structure of pointlight walkers also reduces the evoked eventrelated potentials over the occipitotemporal cortex, but that interference with the temporal structure of the stimuli evoked event-related potentials similar to normal biological motion stimuli. We systematically investigated the influence of spatial and temporal manipulation on 2 common discrimination tasks and compared it with predictions of a neurocomputational model previously proposed. This model first analyzes the spatial structure of the stimulus independently of the temporal information to derive body posture and subsequently analyzes the temporal sequence of body postures to derive movement direction. Similar to the model predictions, the psychophysical results show that human observers need only intact spatial configuration of the stimulus to discriminate the facing direction of a point-light walker. In contrast, movement direction discrimination needs a fully intact spatiotemporal pattern of the stimulus. The activation levels in the model predict the observed eventrelated potentials for the spatial and temporal manipulations.

## Introduction

The human visual system is highly sensitive to the movements of other individuals. Even when the visual information about a person is reduced to only a few point-lights, the depicted figure can be detected within a fraction of a second (Johansson, 1973). The sparse information in these so-called biological motion stimuli is even sufficient to recognize the figure’s gender (Kozlowski & Cutting, 1977; Troje, 2002; Pollick, Lestou, Ryu, & Cho, 2002; Troje, Westhoff, & Lavrov, 2005), to identify individuals (Cutting & Kozlowski, 1977; Loula, Prasad, Harber, & Shiffrar, 2005), and to recognize complex movements (Johansson, 1973; Dittrich, 1993).

**Figure 1.**
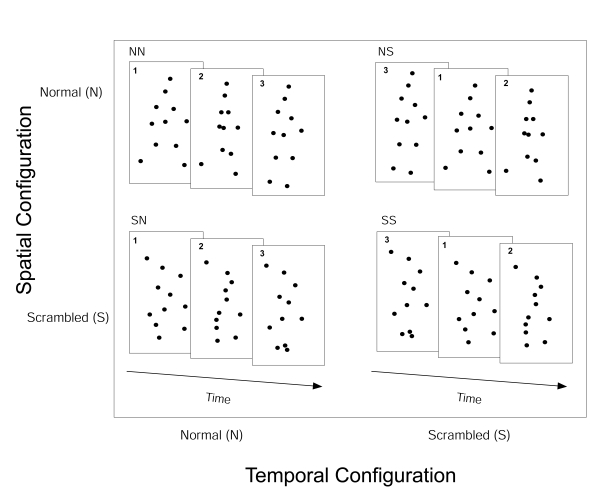
Illustration of the stimuli. In the normal walker, the points are located on the major joints of the body and move with the movement of those joints. In the spatially scrambled stimuli, the dots are initially displaced and then move according to the trajectories of the respective joints at the displaced location. In the temporally scrambled stimuli, each animation frame corresponds to one frame of the temporally normal condition but the order in which the frames are shown is randomized. Combination of these procedures gave four conditions: spatial and temporal configuration intact (Spat:N-Temp:N), spatial configuration intact and temporal configuration (i.e., frame order) scrambled (Spat:N-Temp: S), spatial configuration scrambled and spatial configuration intact (Spat:S-Temp:N), spatial and temporal configuration scrambled (Spat:S-Temp:S).

Because of the speed, accuracy and apparent uniqueness of biological motion-processing, the existence of brain areas specialized for the perception of biological motion has been proposed. Indeed, many studies have reported activation of the superior temporal sulcus (STS) predominantly by biological motion stimuli (Bonda, Petrides, Ostry, & Evans, 1996; Oram & Perrett, 1996; Puce, Allison, Bentin, Gore, & McCarthy, 1998; Grossmann et al., 2000; Beauchamp, Lee, Haxby, & Martin, 2002; Santi, Servos, Vatikiotis-Bateson, Kuratate, & Munhall, 2003; Thompson, Clarke, Stewart, & Puce, 2005) when compared against control stimuli consisting of scrambled biological motion. In spatially-scrambled biological motion the spatial structure of the stimulus is destroyed by the randomizing of the starting positions of each of the dots (see Figure 1) so the motion trajectories of the single dots are intact but the spatial relationships between the dots of the stimulus no longer match the spatial structure of the human body. Such spatial scrambling also reduces event-related potentials (ERPs) observed in response to biological motion stimuli (Hirai & Hiraki, 2006). In the same study, ERPs elicited by temporally scrambled biological motion stimuli were also investigated. In temporally scrambled biological motion the temporal structure of the stimulus is destroyed by the randomizing of the order in which the animation frames are presented (see Figure 1). In this case, the stimulus no longer resembles a walking figure but rather a rapid succession of temporally unrelated body postures. Such temporal scrambling had only a negligible influence on the ERP magnitude, much less than spatial scrambling. Hirai and Hiraki suggested that the results of their ERP study reflect a perceptual effect. Because their subjects, however, viewed the stimulus only passively they could not study perceptual issues. Here we investigate perceptual discrimination tasks with normal and temporally scrambled stimuli.

We have recently proposed a neurocomputational model of biological motion perception from configural form cues (Lange & Lappe, 2006). This model consists of two hierarchically organized stages. The first stage analyzes the spatial structure of the stimulus frames by template matching to a set of body shape templates. The second stage analyzes the temporal arrangement of the body templates. The model is consistent with a wide range of psychophysical and neurophysiological data (Lange & Lappe, 2006; Lange, Georg, & Lappe, 2006). Because of its construction, the first stage of the model should be largely unaffected by the temporal order of the stimulus frames. This stage should therefore work equally well with temporally normal as with temporally scrambled stimuli. In contrast, destroying the configural information by scrambling the positions of the dots would strongly impair the template-matching process and thus the ability of the model to recognize a walker, so perceptual tasks that require only the first stage of the model, such as discrimination of the facing direction of the stimulus, should be unaffected by temporal scrambling, but should be affected by spatial scrambling. In contrast, tasks that involve the temporal order analysis in the second stage of the model should suffer from both temporal and spatial scrambling.

In order to relate behavioural observations to model predictions we employ two perceptual discrimination tasks, namely the discrimination of the facing direction of the stimulus (facing to the left or to the right) and the discrimination of the walking direction of the stimulus (walking forward or backward). These tasks have been previously linked to the two stages of the model (Lange & Lappe, 2006; Lange, Georg, & Lappe, 2006). Like Hirai and Hiraki (2006), we used a complete experimental design, i.e., we manipulated in all tasks the spatial, temporal and combined spatio-temporal configuration of the stimuli. In some cases, for instance, when walking direction has to be judged from stimuli without temporal order, this yields trivial and predictable results for the model and for the behavioural experiment. We report these results, however, for the sake of completeness and because they are still important in the combination of model and psychophysical data, as they provide information on the validity of the model. We show that observers can solve the facing direction task even with temporally scrambled stimuli, similar to the model predictions. We further show that the activation levels in the neural integrators of the model are similar to the ERP results reported by Hirai and Hiraki.

## Methods

### Model

**Figure 2.**
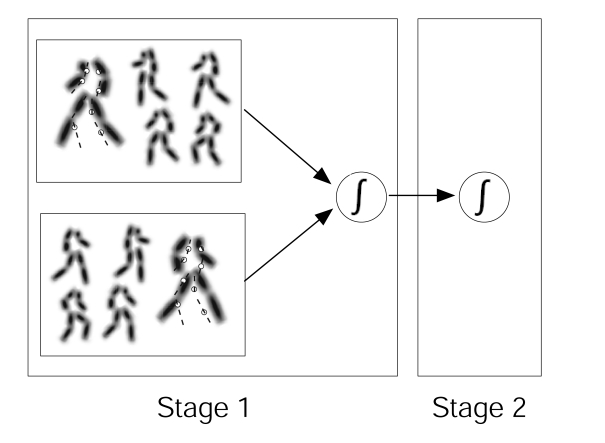
Illustration of the model. The body templates of the model are illustrated as blurry stick-figures and are subdivided into sets for left and right orientation. A stimulus frame is indicated by the white dots (the dashed lines in the stimulus are only for illustration and not in the real stimulus). Stage 1 analyzes only the spatial information of the stimulus by comparing the stimulus dots with static templates of a walker facing either to the right or to the left and feeding the output in a leaky integrator. The outcome of this operator can be read out for the discrimination of the orientation of the figure, or it can be forwarded to a second leaky integrator, which analyzes the temporal information about the stimulus frames (Stage 2). For details about the model see Lange and Lappe (2006).

We first briefly describe the main features of the model (see Lange & Lappe, 2006, for a detailed description). The model used a set of templates which represent static snapshots of a walking human figure. For these templates we recorded the walking movements of nine human persons. We attached sensors to the main joints (i.e. ankles, knees, hips, wrists, elbows and shoulders) and recorded their movements while the subjects walked in a magnetic field generated by two cubes (MotionStar, Ascension). The spatiotemporal signals of the sensors were transmitted to a computer and a walking cycle was divided into 100 static, temporally equidistant frames. From these data we produced line drawings of a walking human person by connecting the single sensor dots in the anatomically correct way. This provided 100 static template frames out of a walking sequence for a walker facing to the right and 100 static template frames out of a walking sequence for a walker facing to the left (see Figure 2). The size of the template frames was normalized to the size of the stimuli.

These template frames are used in the first stage of the model. In this first stage the model analyzes the structural information in each stimulus frame separately. For each stimulus frame the model compares the dot locations in the stimulus frame with all of the 200 templates and computes a distance measure to each template. This matching algorithm computes the shortest Euclidian distance of each single stimulus dot to one of the locations on the template frames and subsequently the sum of all the individual dot distances. The best matching (i.e., least distant) templates from each facing direction set (left or right) are then fed into two leaky integrators. This procedure is repeated for subsequent stimulus frames and the overall matches for left and right facing directions are accumulated in the leaky integrators. The final values of the leaky integrators determine the model decision whether the stimulus belonged to the set for facing to the right or to the set for facing to the left.

In the second stage the model uses the frames selected in Stage 1 to analyze their temporal order. The leaky integrators used in the second stage weigh their inputs depending on whether consecutive frames are recognized as arranged in descending or ascending order. The outcome of these operators are used as decision variables for forward (i.e., frames in ascending order) or backward (i.e., frames in descending order) movement (Figure 2).

In all simulations described below the number of stimulus frames presented to the model was always matched to the number of stimulus frames presented to the human observers in the identical task (i.e., for a frame duration of 30 ms we presented 33 frames, see *Experimental methods* section below).

### Experiments

#### Stimuli

The stimuli are based on a computer algorithm (Cutting, 1978) which artificially simulates the movement of a human body depicted by a few point-lights, viewed from the side. Eleven point- lights were located on the head, both elbows, both wrists, both knees, both ankles and on the midpoint between the shoulders and the midpoint between the hips. All translatory movements were eliminated so that the point-light walker seemed to walk on a treadmill.

 The choice of the artificial stimulus rather than the recorded walking movements of real persons was motivated by two considerations. First, this stimulus was also used in the ERP study by Hirai and Hiraki (2006), with which we want to compare our simulations. Second, since the model uses real walker data as templates use of the same data for the stimuli would always give a perfect fit, since there is always one stimulus and one template frame that are exactly identical. The artificial stimulus is never fully identical to the template and there will always be some mismatch to the templates such that the matching procedure is more demanding. 

We used four different stimulus conditions (see Figure 1): We presented the single, spatially intact, frames of the stimulus sequence in normal order (spatial configuration normal, temporal order normal [Spat:N-Temp:N]) or we randomized the frame order (spatial configuration normal, temporal order scrambled [Spat:N-Temp:S]). Furthermore, we presented the stimuli spatially scrambled but with the correct frame order (Spat:S-Temp:N) or the stimulus was spatially and temporally scrambled (Spat:S-Temp:S). We obtained the spatial scrambling of the stimulus by providing each dot independently with a spatial offset in the range of –2.5° to +2.5°.

#### Subjects

Eight human subjects (five males, including one of the authors; ages 24-37) participated in the psychophysical experiments. They all had normal or corrected-to-normal vision. Four of the subjects (three male; ages 30-37) were experienced in psychophysical tasks using point-light walkers. The other four subjects (ages 24-26) had never before participated in experiments using point-light walkers. These inexperienced subjects viewed three trials of each condition without feedback before the experiment.

#### Experimental methods

The subjects sat in a dimly-lit room, 60 cm in front of the monitor, and viewed the stimulus binocularly. Stimuli were presented on a monitor with a resolution of 1280 x 1024 pixels and a display size of 30 x 40 cm. The monitor refresh rate was 100 Hz. A single stimulus frame was presented for 30 ms (three monitor frames) while the walking speed was 1.0 s per one walking cycle.

The stimulus covered a field of 4° x 2° and consisted of white dots (2 x 2 pixels) on a black background. In each task, the starting-phase in the gait-cycle was randomized, conditions were presented in random order and the stimulus position had a randomly-chosen spatial offset (between 0° and 1° in a horizontal and vertical direction) to avoid spatial cues caused by the position on the screen.

We presented 15 repetitions of each condition in randomized order. Subjects had to indicate their decision in the respective discrimination task by pressing one of two buttons in front of them. After the button press the next stimulus presentation started. Each trial lasted for a maximum of three gait cycles. Subjects were, however, allowed to respond as soon as they recognized the walker, whereupon that trial ended and the next trial started.

#### Tasks

In the facing-direction task, the stimulus walked forward and faced either to the left or to the right. The subject had to report the direction the walker faced (left or right).

In the walking-direction task, the stimulus frames were shown either in normal temporal order (forward movement) or in reverse order (backward movement). Both stimuli comprised exactly the same frames and only their temporal order differed (Beintema, Georg, & Lappe, 2006). Subjects had to report the walking direction of the stimulus (forward or backward). No feedback was given in any task.

 For all tasks we used the artificial stimulus based on the algorithm by Cutting (1978), as did Hirai and Hiraki (2006). Especially for the facing-direction task it is important to note that in this stimulus all dots presented in a single trial are symmetrically distributed around the vertical axis. In contrast, for natural walking persons this axis is tilted in the walking direction. By using the artificial stimulus we prevented the human subjects from using the slant as a cue to solve the task.

## Results

### Behavioural data

**Figure 3.**
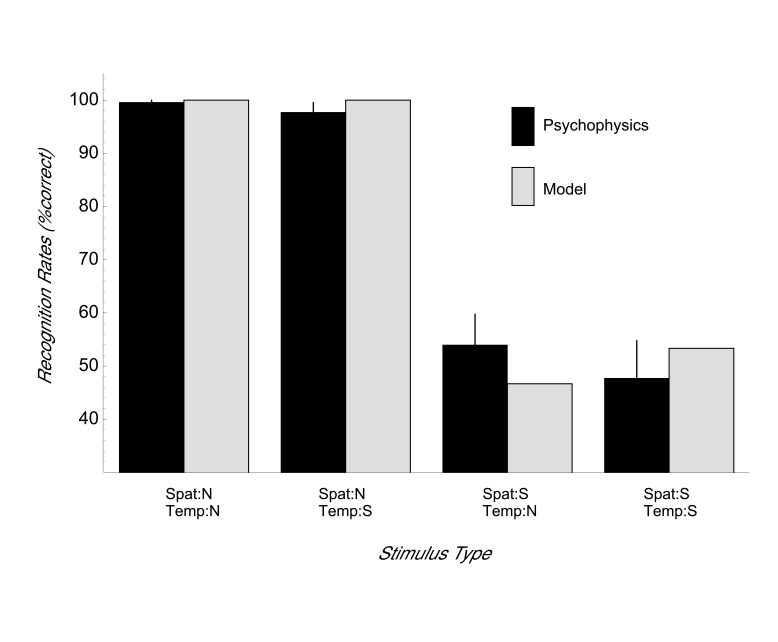
Results of the orientation task for human subjects and model Stage 1 for the four stimulus types. Psychophysical data are presented as mean ± 1 standard error of the mean.

Figure 3 shows the results of psychophysical experiments along with model predictions derived from computer simulations with identical stimuli for the facing-direction task. The model predicts that the facing direction can be discriminated independent of the temporal order of the stimulus frames as long as the spatial configuration of the point-lights within one stimulus frame is intact (recognition rates 100% for conditions Spat:N-Temp:N and Spat:N-Temp:S) (see Figure 3). If the configural arrangement of the dots is destroyed, correct discrimination is impossible and recognition rates drop to a level around chance (47% for condition Spat:S-Temp:S and 53% for condition Spat:S-Temp:N).

The human subjects discriminated the facing direction of the stimulus reliably when the spatial configuration was intact (conditions Spat:N-Temp:N and Spat:N-Temp:S), but were unable to discriminate the facing direction when the spatial configuration was destroyed (Spat:S-Temp:N and Spat:S-Temp:S). For a statistical analysis of the psychophysical results, we calculated a 2 x 2 x 2 ANOVA with repeated measures and including the factors spatial scrambling (normal/scrambled), temporal scrambling (normal/scrambled), and subjects (experienced/inexperienced).

The main factor spatial scrambling revealed a highly significant effect, *F*(1, 3) = 310.1,*p* < .001, i.e., mean recognition rates for spatially normal stimuli were higher than for spatially scrambled stimuli (98.4% and 50.8%, respectively). In contrast, there were no statistically significant effects for the main factor temporal scrambling, *F*(1, 3) = 0.3, *p *= .62 (mean for temporally normal stimuli 76.6%, for temporally scrambled stimuli 72.7%), or for the main factor subject, *F*(1, 3) = 0.2, *p *= .72 (mean experienced 75.8%, inexperienced 73.4%). Furthermore, there were no significant effects for the interactions of the factors: subject-spatial scrambling, *F*(1, 3) = 0.2, *p *= .72; subject-temporal scrambling, *F*(1, 3) = 0.6, *p *=.49; spatial-temporal scrambling, *F*(1, 3) = 0.1, *p *= .76; subject-spatial-temporal scrambling, *F*(1, 3) = 0.1, *p *= .77. The lack of interaction between spatial and temporal scrambling indicates that the decrease of performance observed for spatial scrambling is similar for temporally normal and scrambled stimuli.

**Figure 4.**
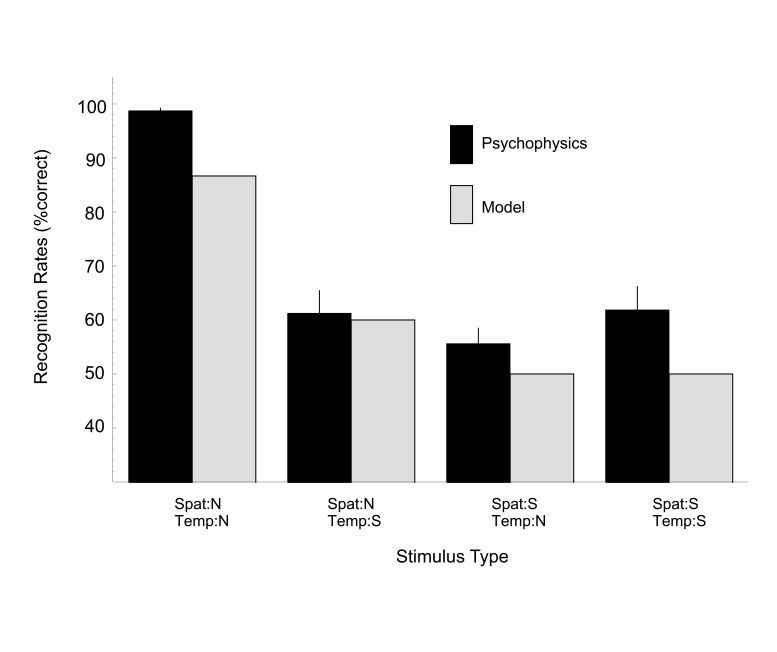
Results of the forward/backward task for human subjects and model Stage 2 for the four stimulus types. Psychophysical data are presented as mean ± 1 standard error of the mean.

For the forward/backward task (see Figure 4), the model predicts that the task can be solved only if the temporal and spatial configurations of the stimulus are intact. Recognition rates for fully intact stimuli are at 87% whereas the recognition rates for all other conditions are around chance level.

In agreement with the model predictions, subjects were able to solve the task only if spatial and temporal configurations were normal (recognition rates for condition Spat:N-Temp:N were 99%). If only the spatial or the temporal component is impaired, the task is no longer solvable and the recognition rates drop to chance level (see Figure 4).

Consequently, a statistical analysis (2 x 2 x 2 factorial design, see above) revealed significant effects for spatial scrambling, *F*(1, 3) = 113.7,*p* < .01. There were no significant effects for the factor temporal scrambling, *F*(1, 3) = 7.7, *p *= .07, or for the factor subjects, *F*(1, 3) = 0.05, *p *= .83. The interaction between spatial and temporal scrambling, however, was significant, *F*(1, 3) = 22.2, *p *= .02, indicating that the influence of temporal scrambling was different for spatially normal and scrambled stimuli. All other interactions revealed no significant effects: subject-spatial scrambling, *F*(1, 3) = 2.4, *p *= .22; subject-temporal scrambling, *F*(1, 3) = 3.6, *p *= .16; subject-spatial-temporal scrambling, *F*(1, 3) = 1.2, *p *= .36.

### Comparison with neural activities

**Figure 5.**
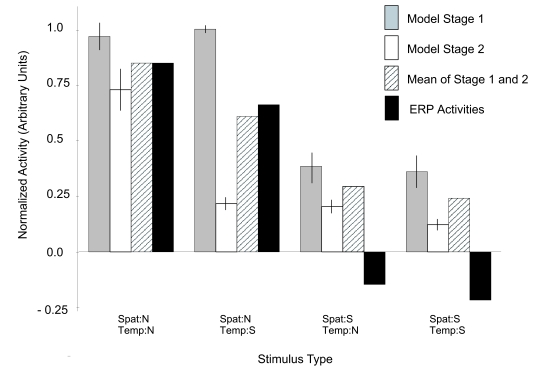
Simulated activity of model Stage 1 (grey bars) and Stage 2 (white bars) and the mean over both stages (shaded bars). The model predictions were compared with activities (black bars) obtained in an ERP study (Hirai & Hiraki, 2006). Model data of Stages 1 and 2 are presented as the mean activities of seven simulations ± 1 standard error of the mean.

 We evaluated the relative output activities of the two model stages to the four different types of stimuli and compared them with ERPs reported by Hirai and Hiraki (2006). We presented the stimuli to both model stages and calculated the maximum output of these stages to each stimulus. The procedure followed in detail that used for predicting fMRI activities in Lange and Lappe (2006). The results are shown in Figure 5. The model predicts that there is no significant activity difference between temporally normal and temporally scrambled stimuli in model Stage 1, as long as the stimuli are presented in spatially normal configuration. Statistical analysis (2 x 2 factorial design with the spatial and temporal configuration as factors, see above) revealed a highly significant effect for spatial scrambling, *F*(1, 6) = 155.7,*p* < .01, but no significant effects for the factor temporal scrambling, *F*(1, 6) = 0.003, *p *= .96, or for the interaction between spatial and temporal scrambling, *F*(1, 6) = 0.3, *p *= .61. 

Statistical analysis for the activities of model Stage 2 revealed highly significant effects for spatial scrambling, *F*(1, 6) = 33.8,*p* < .01, and for temporal scrambling, *F*(1, 6) = 42.3,*p* < .01. Furthermore, a statistically significant interaction existed between spatial and temporal scrambling, *F*(1, 6) = 32.4, *p* < .01, indicating that the influence of scrambling is different for spatially and temporally normal stimuli.

 We thus conclude that both temporal and spatial scrambling reduce the neural activity in Stage 2 whereas only spatial scrambling reduces the activity in Stage 1. A quantitative comparison of the amount of activity reduction between the model and the ERP data from Hirai and Hiraki (2006) encounters two problems, however. First, Hirai and Hiraki analyzed ERP amplitudes for the sensors T5 and T6. These sensors are in the proximity of the STS region but may also include averaged signals from brain areas in the temporal cortex around STS. The relative weighting of these contributions is not known. For the comparison we therefore decided simply to average the responses of both model stages, since we reasoned that both Stage 1, which correlates with areas like the fusiform face area (FFA), the occipital face area (OFA) or the extrastriate body area (EBA) (Lange & Lappe, 2006), and model Stage 2, which correlates with STS (Lange & Lappe, 2006), may be included in the ERP signal. Second, there is obviously an arbitrary scaling involved between the ERP signal, measured in mV, and the model activity, which is essentially a number between 0 and 1 and cannot be negative. We decided simply to scale the model activity for the condition Spat:N-Temp:N to the respective ERP value. This allows a qualitative comparison with the drop in the other conditions. We then compared the results from the model activations to the averaged ERP amplitudes reported for the T5 and T6 sensors by Hirai and Hiraki. 

 For the averaged responses, the model predicts that the amplitude of the condition Spat:N-Temp:S has about 70% of the amplitude of condition Spat:N-Temp:N, whereas the other two conditions, which reflect spatially scrambled configurations, elicit only about 30% of the responses. Similarly, Hirai and Hiraki (2006) report that the condition Spat:N-Temp:S still elicits 80% of the amplitude of condition Spat:N-Temp:N whereas the magnitude of the response to the spatially scrambled conditions Spat:S-Temp:N and Spat:S-Temp:S is significantly smaller (see Figure 5). 

## Discussion

We investigated how manipulations of the temporal and spatial configuration of a point-light walker affect the discriminability of particular aspects of biological motion. We tested the influence of spatio-temporal stimulus properties of biological motion by comparing the predictions of a computational model with the results from behavioural tasks and with results obtained from a previous study measuring event-related potentials (Hirai & Hiraki, 2006). The results provided a behavioural correlate and an explanation from a computational viewpoint of the results of the ERP study. Furthermore, the results of the experimental and computational approach demonstrate the task-dependent use of information in biological motion processing: Spatial but not temporal information plays an important role in detecting a walker’s facing direction, but both spatial and temporal information are important for walking direction discrimination.

First, we tested the influence of spatio-temporal manipulations if the task was to report the facing direction of a point-light walker. As predicted by the model, recognition rates in the facing-direction task depended on the spatial rather than on the temporal structure. Since only the first stage of the model is used for the facing-direction task, and since the first stage treats single stimulus frames independently, the results obtained for the model are not surprising as they could be qualitatively predicted from the model configuration. The implications of these data, however, are not trivial. From the psychophysical point of view it is not obvious that the facing-direction task can be solved even if the frame order is randomized. The psychophysical experiments confirmed the model predictions that only form information and no temporal or motion signals are necessary to solve the facing-direction task. These results were independent of the level of experience of the subjects. Both experienced and inexperienced subjects reliably discriminated the facing direction of the walker in the temporally scrambled condition. It is furthermore interesting to note that discrimination of the facing direction did not require a clear percept of a walking figure. Both experienced and inexperienced subjects reported that they had no clear percept of a walking human person in the condition Spat:N-Temp:S but that they did perceive the structure of a human body. Apparently, this coarse information is sufficient to solve the facing-direction task. This is consistent with the proposed two-stage procedure of the model.

 These results cannot be explained by models that emphasize local motion analysis. For instance, the model of Giese and Poggio (2003) contains a “form” and a “motion” pathway. Classical point-light stimuli, such as the Cutting (1978) walker used here and in Hirai and Hiraki (2006), activate only the motion pathway and the form pathway does not respond to point-light stimuli (see Figure 5, see also Giese & Poggio, 2003, p. 186). Thus, point-light walkers are only processed in the motion pathway of that model. Local motion signals or “opposing motion vectors” (Casile & Giese, 2005) are essential for this model to extract information about a point-light stimulus. Temporal scrambling eliminates these local or opposing motion signals and would destroy responses in the model. Furthermore, Giese & Poggio have shown that the high level motion pattern neurons in their model produce activity only when the stimulus frames are presented in correct order. If the frames are presented in randomized order, the activity drops to baseline. This is similar to the second stage in our model, but because decisions on the facing direction in our model are derived from the first stage, which analyzes body form from point-light stimuli, our model correctly predicts performance in the facing-discrimination task with temporally scrambled stimuli. 

The similar results of model and human observers suggest a similar strategy to solve the task, namely to analyze the facing direction of the walker in each frame independently and then integrate this information into an overall judgement about the facing direction of the stimulus. For the condition Spat:N-Temp:S, however, it might be possible that subjects do not treat each frame independently from the others but first integrate the 33 frames of the stimulus to a coherent structure and then judge the facing direction based on this information. Given the similar results of the model and the subjects in all tasks, it seems likely that subjects and model share common strategies to solve them (i.e., the way the model solves the task – by analyzing the dynamic structure of the stimulus frames). Nevertheless, even the second strategy explained above would suggest that subjects can solve the facing-direction task solely on the basis of information about the structure without the need of motion or temporal information. This conclusion is in line with the conclusion drawn from the strategy of the model: The facing-direction task can be solved by only analyzing information about the structure.

 Troje and Westhoff (2006) reported that human observers are able to discriminate the facing direction of spatially scrambled point-light displays above chance level. In our study, subjects were unable to report the facing direction of a spatially scrambled stimulus. This seemingly contradictory observation may be explained by the different stimuli used in the two studies. While Troje and Westhoff used stimuli recorded from movements of human walkers, our experiments, and those of Hirai and Hiraki (2006), used the artificial stimulus developed by Cutting (1978). The limb movements of this artificial stimulus are more symmetric than those of a real walker. This reduces the possibility of using the asymmetries of certain limbs (such as the feet) to infer walking direction and focuses the task on global aspects of the body configuration. Since in condition Spat:N-Temp:S the symmetric artificial stimulus allowed easy facing direction we think the stimulus is well suited to study global aspects of biological motion processing. 

 The differences, however, between our results for spatially scrambled walkers and those of Troje and Westhoff (2006) reveal that humans can use different strategies to solve the facing-direction task. Discrimination of walking direction might be achieved either by a global, holistic analysis of the entire human body or subjects might pick out specific stimulus dots that provide cues for a specific task, for example asymmetric trajectories during a walking cycle such as the feet for a discrimination of walking direction (Troje & Westhoff, 2006; Mather, Radford, & West, 1992; Lange et al., 2006). It is, however, unclear whether specific, local cues provide enough information for the perception of a human body, that is for tasks beyond a discrimination task. For example, Pinto and Shiffrar (1999) challenged the view that the extremities of the human body alone provide sufficient information to recognize a human body. In their study, observers were instructed to report freely descriptions of the stimulus, which was either a point-light display of the entire human body, of different subconfigurations (e.g., only the left or the right side of the body), or of a spatially scrambled version of the whole-body point-light display. For the subconfigural views of the stimulus, the observers reported seeing a human body nearly as often as they did for the whole stimulus displays. In contrast, the responses to the randomly-located limbs differed significantly from the responses to the whole-body representations. Pinto and Shiffrar concluded that “configural information is specifically indicative of human form in the perception of biological motion displays” (p. 313). Single stimulus dots might therefore propose information to solve a facing-direction task because of their asymmetric trajectories. It seems unclear, however, whether the results of such discrimination tasks provide insights into the perception of an entire walking human body. 

Our results reveal that subjects can solve the task by using a different strategy. Instead of exploiting information about single dots or limbs they could solve the task by judging the structure of the walker. For this the feet might also be important, but because they give the most information about the structure and not because of their asymmetric movements (Lange et al., 2006). When subjects use this strategy, they do not need the correct movement of the human body, so that even if subjects exploit this information the question of how humans perceive the movement of a human body may be only partially answered by the facing-direction task.

In contrast, when the task was to discriminate walkers moving forwards or backwards, the model predicted that manipulation of the temporal stimulus configurations had a strong influence on the recognition rates. Likewise, the subjects could solve this forward/backward task only if the spatio-temporal configuration of the stimulus was intact. The results with respect to temporal scrambling are trivial since the temporally scrambled stimulus does not carry any information about the walking direction. Nevertheless, we felt it important to include this task in the study because the results in the spatially scrambled condition are not trivial. Purely spatial scrambling keeps the order of frames intact but because the spatial scrambling interferes with the template-matching process in model Stage 1 the discrimination performance of the model is disrupted. Likewise, spatial scrambling alone disrupted discrimination performance for walking direction in our human subjects. Our results thus revealed that in contrast to the facing-direction task the forward/backward task demands the entire and intact spatio-temporal configuration of the stimulus, so this task seems better suited to investigate the perception of a walking human.

 The second focus of our study refers to the question which brain areas process the relevant information of the stimuli. It is clear that the STS is critically involved in the perception of biological motion (e.g., Bonda et al., 1996; Grossmann et al., 2000; Thompson et al., 2005). However, it is less clear what information processing steps occur until the information reaches the STS. While some studies claim a crucial influence of areas that are classically assigned to motion perception (e.g., Giese & Poggio, 2003; Peuskens, Vanrie, Verfaillie, & Orban, 2005) other studies challenge this view (e.g., Grossman, Batelli, & Pascual-Leone, 2005) or emphasize the role of areas which are thought to process static images and forms (e.g., Grossman & Blake, 2002; Michels, Lappe, & Vaina, 2005; Jokisch, Daum, Suchan, & Troje, 2005). Hirai and Hiraki (2006) measured ERP amplitudes when subjects passively viewed point-light displays. They demonstrated that biological motion displays induce brain activation measured by electrodes over the occipital temporal cortex even when the temporal structure of a point-light walker is destroyed. In a previous study we assigned the first stage of our model to form processing areas like FFA, OFA, and EBA and the second stage to STS (Lange & Lappe, 2006). The average over the activation in these stages predicts the results observed in the ERP study by Hirai and Hiraki and provides a natural explanation for the activation in the temporally scrambled conditions. Note that the model is not suited to reproduce data quantitatively from ERP studies. Rather, it is suited to predict qualitatively whether a decrease of neural activity should be expected or not. 

 We found, however, that the importance of the temporal structure depended on the task. If subjects were asked to judge the walking direction in two stimuli that comprised exactly the same stimulus frames (but presented in different temporal orders), the results relied on the spatial as well as on the temporal structure of the stimulus. In the study by Hirai and Hiraki (2006) subjects viewed the stimulus passively without explicitly attending to a task. It is possible that the subjects solely attended to the human structure irrespective of whether this figure walked in an articulated way. Similarly, in our facing-direction task subjects solely needed structural information to solve the task. For the forward/backward tasks we found that destroying the temporal structure eliminated the ability to solve the task. It would therefore be interesting to investigate whether task dependencies also exist in the ERP signal, as predicted by our model. Recent studies have demonstrated that attention (Hirai, Senju, Fukushima, & Hiraki, 2005; Pavlova, Birbaumer, & Sokolov, 2006) and the task (Vaina, Solomon, Chowdhury, Sinha, & Belliveau, 2001) can modulate brain activity when subjects view biological motion stimuli. It would be interesting to see whether the ERP responses for identical stimuli but different tasks would be modulated by the active role of the viewer rather than by the passive bottom-up analysis of the stimulus. 

 The results of our psychophysical experiments and the model simulations imply that biological motion is processed by spatio-temporal sampling of form information. Depending on the task, however, different information is emphasized differently. In models that analyze the local motion signals in the stimulus (e.g., Giese & Poggio, 2003) the scrambled temporal order will elicit activation levels much smaller than those of stimuli with correct temporal order. Such models therefore cannot account for the results presented in the ERP study by Hirai and Hiraki (2006) nor can they model the psychophysical data presented in our study. In contrast, a model that analyzes global form information and then integrates the global form information temporally can predict the results in our study and would predict the results by Hirai and Hiraki. Whether the results presented in this study can be extended to other types of biological motion stimuli remains to be investigated. In the present study, however, we found that temporal information might be redundant and will only be used if it is essential to solve the task. 
